# Long-Term Imaging of *Caenorhabditis elegans* Using Nanoparticle-Mediated Immobilization

**DOI:** 10.1371/journal.pone.0053419

**Published:** 2013-01-03

**Authors:** Eric Kim, Lin Sun, Christopher V. Gabel, Christopher Fang-Yen

**Affiliations:** 1 Department of Bioengineering, University of Pennsylvania, Philadelphia, Pennsylvania, United States of America; 2 Department of Physiology and Biophysics, Boston University School of Medicine, Boston, Massachusetts, United States of America; Harvard University, United States of America

## Abstract

One advantage of the nematode *Caenorhabditis elegans* as a model organism is its suitability for *in vivo* optical microscopy. Imaging *C*. *elegans* often requires animals to be immobilized to avoid movement-related artifacts. Immobilization has been performed by application of anesthetics or by introducing physical constraints using glue or specialized microfluidic devices. Here we present a method for immobilizing *C. elegans* using polystyrene nanoparticles and agarose pads. Our technique is technically simple, does not expose the worm to toxic substances, and allows recovery of animals. We evaluate the method and show that the polystyrene beads increase friction between the worm and agarose pad. We use our method to quantify calcium transients and long-term regrowth in single neurons following axotomy by a femtosecond laser.

## Introduction

*C. elegans’* small size and optical transparency make it unique among multicellular model organisms in that microscopy can be performed throughout the intact, live adult animal. Important experiments and techniques enabled by the worm’s transparency include the mapping of its developmental cell lineage [1], laser ablation of cells and nerve fibers [2], and imaging of fluorescent protein tags [2]. Imaging at high spatial resolution generally requires *C. elegans* to be at least partially immobilized, because animal movements impede identification of cells and cause movement artifacts. Immobilization is usually performed using anesthetic agents such as sodium azide, phenoxypropanol, and levamisole [3]. However, these compounds are often not compatible with the physiological processes being studied and usually preclude long-term imaging.

An alternative immobilization method used for calcium imaging [4] and electrophysiological [5] studies has been to glue the worm onto a substrate using cyanoacrylate adhesive. Gluing has the advantage of preserving short-term physiological function and allowing physical access to the animals, for example to deliver sensory stimuli or introduce electrodes. Limitations of glue immobilization include the technical difficulty of the procedure, the inability to recover the animals, and optical distortion from the highly refractile glue.

Several groups have described microfluidic devices for immobilization and imaging of *C. elegans*. These microfabricated devices containing elastomeric channels and chambers have been used to immobilize *C. elegans* by compression [6], vacuum suction [7], temporary cooling [8], or carbon dioxide [9]. Some of these microfluidic systems are also capable of automated or semi-automated worm imaging and sorting. However, the cost and technical complexity of microfluidic systems are impediments for many researchers.

Here we describe and characterize a simple method for *C. elegans* immobilization using agarose pads and polystyrene nanoparticles.

## Methods

**Figure 1:**
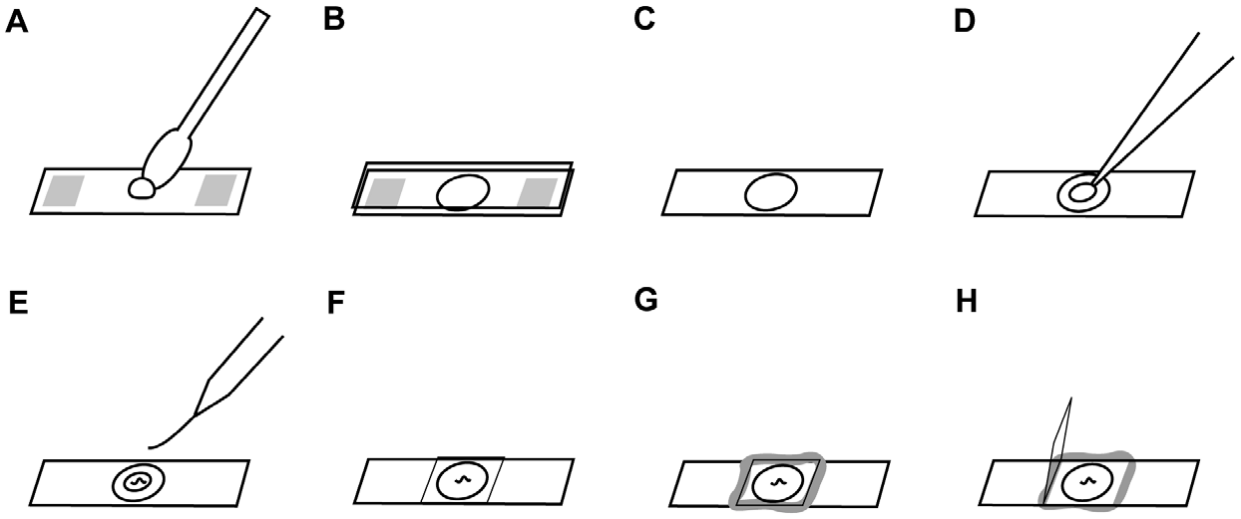
Nanoparticle-mediated immobilization. (a) Transfer molten agarose to slide containing plastic spacers (shown in gray). (b) Quickly add second slide to form pad. (c) After agarose has cooled, remove upper slide and spacers. (d) Add polystyrene nanoparticles. (e) Add washed worm(s). (f) Add coverslip. The worms are now ready for imaging. (g) Seal coverslip with wax if necessary. (h) To recover animals, lift coverslip upwards without sliding.

To immobilize worms (Fig. 1), we placed one or more NGM-washed *C. elegans* and 0.25–2 µL of a suspension of polystyrene beads (Polysciences, 2.5% by volume, 0.1 µm diameter or as specified) on a pad containing 5–10% agarose in NGM buffer (as described [10] but without agar, peptone, or cholesterol) or M9 [10]. We did not find the exact thickness or solute composition of the pad to be important for successful results. To immobilize small larvae it is critical to minimize the volume of fluid on the pads. When immobilizing L1 animals we added only 0.25 µl of the bead solution and allowed uncovered pads to dry for a few minutes before adding worms. To prepare agarose pads we followed a procedure similar to standard methods [11] except using pieces of polyester or PETG sheet with defined thicknesses (McMaster-Carr 9513K42) as spacers instead of layers of tape. For long-term imaging, the cover glass was sealed with a 1∶1 mixture of paraffin and petroleum jelly to prevent dehydration.

To recover worms after immobilization, we lifted one edge of the coverslip from the pad without sliding and added a few µl NGM or M9 buffer to the worms. Worms can then be transferred to OP50-seeded plates using a platinum worm pick or mouth pipette. We found that after 1 hr immobilization approximately 90% of worms can be recovered successfully.

To measure locomotory frequency, we placed worms in a 10 µL droplet of either NGM buffer containing 0.1% BSA or a 2.5% v/v suspension of 100 nm diameter beads, then recorded video sequences of the worms under 10X magnification and bright field illumination using a Leica DM2500P microscope. Videos were recorded to a PC computer at 30 frames per second using a CCD camera (Imaging Source 31BU03.H) and Image Capture software. We measured each worm successively in both NGM and bead suspension; the order of testing was reversed for half the worms. We measured undulatory frequencies by reviewing videos and recording the number of frames required for 10 locomotory cycles during periods of forward movement.

To quantify the degree of immobilization, we used either N2 animals or strain PX437 (gift of B. Gaertner), which expresses the fluorescent calcium indicator YC3.60 in the two AFD neurons under the *gcy-8* promoter. Worms were placed on 5–10% agarose in NGM buffer with or without polystyrene beads. For N2 worms we acquired bright field time lapse microscopy sequences at 10 frames per second using on a Leica DMLB upright microscope with a 10X objective. We used a custom MATLAB program to manually mark the location of the center of the base of the buccal cavity in each image (i.e. the anterior tip of the pharyngeal lumen). For PX437 worms we acquired time lapse image sequences of the fluorescence of either the AFDL or AFDR cell bodies at 100X magnification using a Leica DM2500P microscope in epifluorescence mode using a GFP filter cube (YC3.60 contains CFP and YFP variants but can be readily imaged using GFP optics). For each worm we recorded images every 10 s for 1 hr. We used custom MATLAB software to threshold each image and determine the coordinates of the centroid of the neuronal cell body for each frame. Results for AFD movement and buccal cavity movement are not directly comparable because the buccal cavity tended to move much more than the more posterior AFD cell body.

To measure dorso-ventral widths of freely moving or immobilized worms, we recorded images of worms under bright field illumination at 60X magnification and used ImageJ to measure the distance between dorsal and ventral edges at mid-body.

For laser ablation experiments, we used a 1∶1 dilution of 0.1 µm diameter polystyrene beads in NGM. A 6 µl droplet was placed on a 10% agarose pad to mount 10–15 young adult animals. The additional suspension volume helped to mitigate drying of the pad while mounting multiple animals and during long-term imaging. For extended time-lapse imaging, it was critical to completely encase the agarose pad in the paraffin-petroleum jelly mixture such that it filled as much of the volume under the cover slip as possible. This provides mechanical stability to the preparation over long time periods and was accomplished by injecting the warm paraffin mixture under the cover slip with a pipette.

Laser axotomies were performed using a femtosecond pulsed infrared laser (Coherent Inc.) and a 60X, 1.4 NA objective as described previously [12]. We acquired images using an automated computer controlled microscope system (Nikon) that allowed for time-lapse imaging of multiple (∼15) worms in parallel. Images were initially acquired as z-stacks (10 images in 1 µm steps) and then compressed to generate a single image at each point. Regenerative outgrowth lengths were measured by summing the contour length of all new neuron outgrowths sprouting from either the cell soma or the region near the lesion point. We performed calcium measurements using a strain expressing cameleon YC3.60 in the six mechanosensory neurons and using FRET based ratiometric imaging techniques described previously [13].

## Results

### 1. Degree of Immobilization Depends on Agarose Concentration, Worm Developmental Stage, and Bead Size

**Figure 2:**
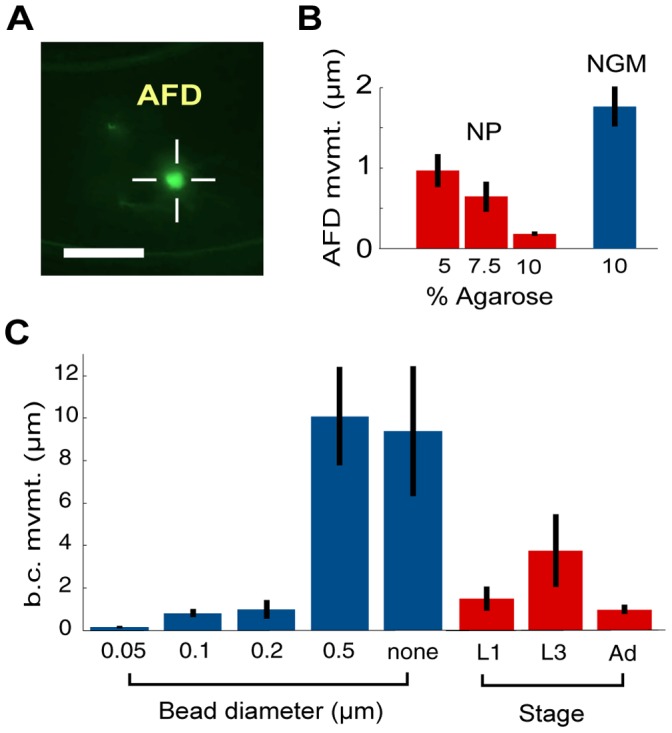
Quantifying movement during immobilization. (a) Fluorescence of AFD soma in a nanoparticle-immobilized *Pgcy-8*::YC3.60 worm. Centroid of cell body indicated by crosshairs. Scale bar: 20 µm. (b) NP: Mean absolute displacement of AFD neuron cell body during 10 s intervals in animals immobilized with 0.1 µm diameter nanoparticles, as a function of agarose concentration in pad. NGM: no beads. (c) Mean absolute displacement of buccal cavity during 10 s intervals for adult worms on 10% agarose pads and varied bead diameters; L1, L3, and adult (Ad) worms immobilized with 0.1 µm beads on 10% agarose pads.

To quantify the degree of immobilization in varying agarose concentrations, we measured the movement of a single AFD neuron (Fig. 2a), located in the head of an adult worm immobilized with 0.1 µm diameter beads, over a 1 hr recording. We measured the coordinates of the cell body’s centroid in each frame, and calculated the average displacement of the centroid between consecutive images acquired 10 s apart.

The movement of the AFD neuron (Fig. 2b) decreased with agarose concentration, from 0.97±0.21 µm for 5% agarose to 0.18±.02 µm for 10% agarose (all values given as mean ± SEM). When worms were prepared on a 10% agarose pad with an equal volume of NGM replacing the bead solution, we found that movement of the AFD neuron (1.8±0.2 µm) was about 10 times greater than the movement in the presence of beads. Therefore beads contribute to immobilization of the worms.

To determine the effect of bead size on worm immobilization we applied the simpler protocol of tracking movement of the buccal cavity using N2 animals. We compared the immobilization of adult worms placed on 10% agarose pads with 0.05 µm, 0.1 µm, 0.2 µm, 0.5 µm, and no beads (NGM buffer). We found that worms immobilized using the smallest (0.05 µm) beads showed the smallest residual movement (0.15±0.05 µm) (Fig. 2c). Immobilization on 0.1 µm and 0.2 µm beads was comparable. 0.5 µm beads were ineffective at immobilizing worms, as they performed similarly to a buffer containing no beads.

To compare immobilization of larval and adult worms, we tracked buccal cavity movements of L1 (first-stage larvae), L3 (third-stage larvae) and adult worms immobilized using 10% pads and 0.1 µm diameter beads. We found that all three stages can be immobilized, but adult worms exhibit somewhat smaller movements compared with L1 or L3 animals (Fig. 2c).

### 2. Dorso-ventral Width Increases with Agarose Concentration

**Figure 3:**
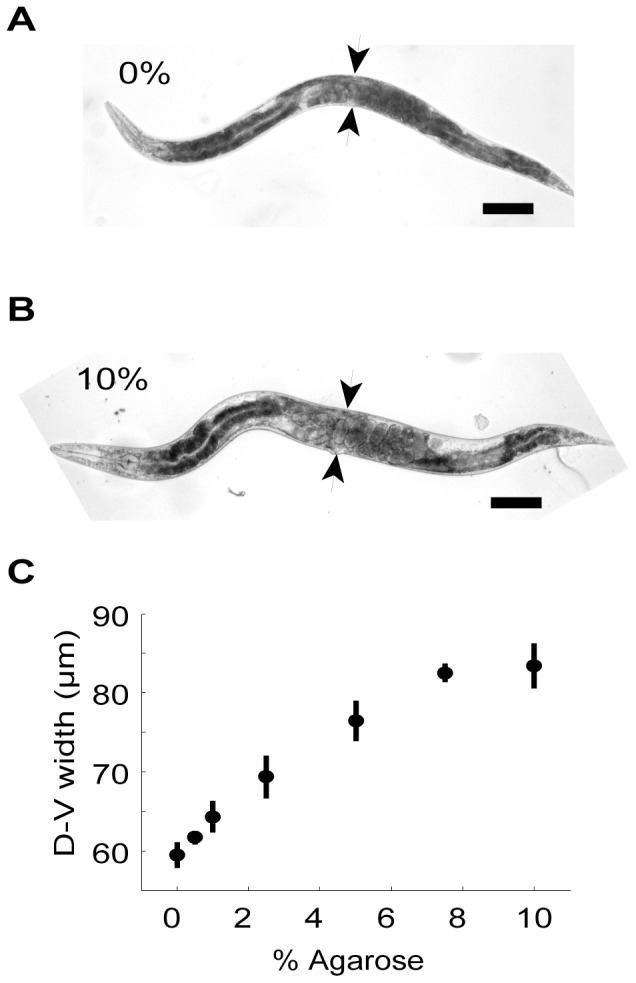
Compression during immobilization. (a) Adult hermaphrodite in NGM buffer. (b) Same animal during immobilization on 10% agarose pad. Arrows indicate measurement of dorso-ventral width. Scale bars: 100 µm. (c) Dorso-ventral width as a function of agarose concentration for 0.1 µm diameter nanoparticle-immobilized worms (n>5 for each point).

We observed that worms appeared wider when immobilized. We measured the distance between the dorsal and ventral edges of the worm (dorso-ventral width) at mid-body as a function of agarose concentration (Fig. 3a–c), using 0.1 µm beads. For 0% we placed worms in a droplet of NGM buffer solution between two slides spaced 500 µm apart. We found that the dorso-ventral width increased with agarose concentration. For 10% agarose the dorso-ventral width was w = 83.4±2.9 µm, approximately 40% greater than the value at 0% (59.4±1.6 µm). The increase in width is consistent with an increased stiffness of the agarose pad, and therefore greater compression of the worm’s elastic body.

### 3. Beads Increase Friction between the Worm and the Pad and/or Coverslip

We sought to understand how polystyrene beads contribute to the animals’ immobilization. One possibility is that the beads increase friction coefficients between the worm and the pad and/or glass it is in contact with (here called the friction hypothesis). Another possibility, not exclusive of the first, is that beads affect the worm’s behavior via an anesthetic or quiescence-inducing effect (here called the anesthetic hypothesis).

To test these possibilities, we evaluated the effects of nanoparticles on the frequency of the worm’s locomotory undulations in the presence or absence of solid surfaces. Undulation frequency reflects both a worm’s level of locomotor activity [14] and the amount of external mechanical resistance [15]. According to standard descriptions of friction, the maximum force resisting a worm’s movements is the product of *(i)* the normal force exerted on the worm by the pad and coverslip and *(ii)* the sum of the friction coefficients between the worm and pad, and between the worm and cover slip.

**Figure 4:**
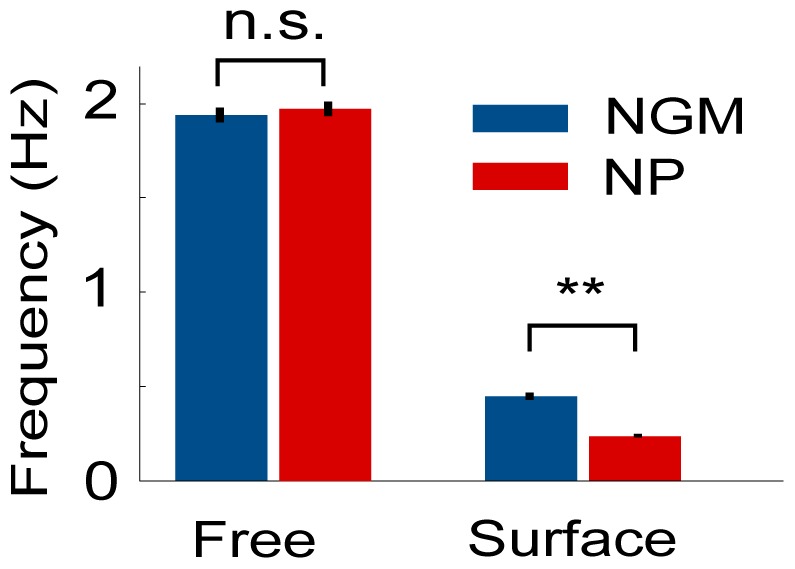
Nanoparticles reduce locomotory rate in a surface-dependent manner. Locomotory frequency of freely swimming worms and worms in contact with 0.5% agarose pads, in the presence (NP) and absence (NGM) of nanoparticles. n = 30 for each group. **p<0.001.

The friction hypothesis predicts that worms moving in contact with solid surfaces will move more slowly with beads than without. Indeed, the average locomotory frequency of worms in 0.5% agarose pads with 0.1 µm beads, 0.23±0.01 Hz was about half that of worms without beads, 0.45±0.02 Hz (Fig. 4).

The friction hypothesis predicts that for worms moving *without* contact with solid surfaces, the normal force is zero and therefore locomotory frequency is independent of friction coefficients (i.e. independent of the presence of beads). Indeed, we did not find a significant difference in frequency between worms placed in a 2.5% v/v suspension of 0.1 µm polystyrene beads, f = 1.97±.04 Hz and worms in NGM buffer, f = 1.94±.04 Hz (p = .48 by Student’s t-test, n = 30). This also suggests that beads do not have an anesthetic effect. Our findings are consistent with a model in which beads increase friction coefficients between the worm’s body and the agarose pad and/or glass cover slip.

### 4. Nanoparticle Immobilization Enables Long-term *in vivo* Imaging of Axon Regeneration

*C. elegans* has emerged as an important model for the study of nerve damage and regeneration, through laser severing of specific nerve processes within an intact animal [3], [16]. We used our method to immobilize worms during laser axotomy and long-term imaging of axonal regeneration.

Cellular calcium signaling is known to play an important role in the neuronal response to damage [17], [18], as well as in growth cone guidance and outgrowth [19]. However, little is known of the localized intracellular calcium dynamics that occur *in vivo* within an injured neuron and how they might initiate and modulate regeneration at different locations within the cell. By measuring both local regenerative outgrowth and the corresponding local calcium signal within laser damaged neurons *in vivo*, we aim to understand how physiological response can initiate regenerative repair on a subcellular level.

**Figure 5:**
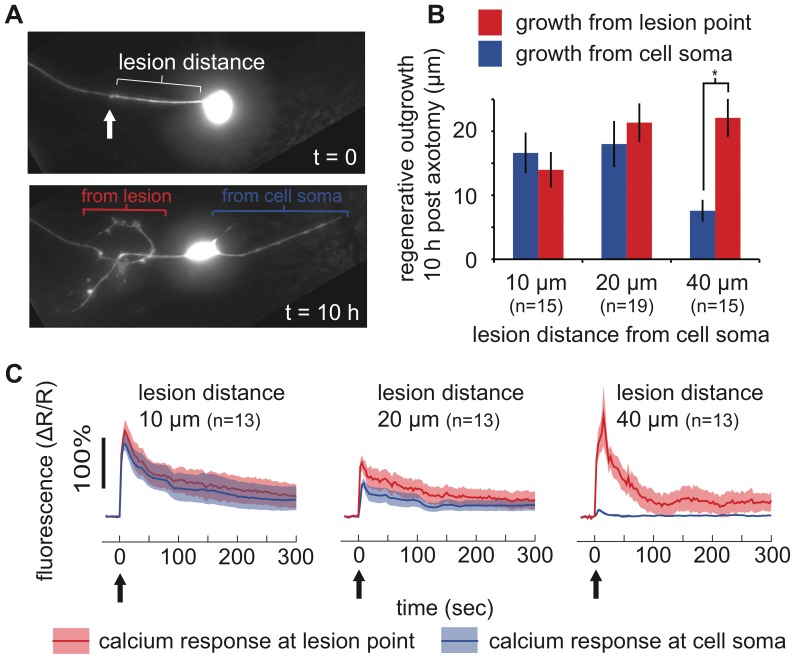
*In vivo* laser axotomy and time-lapse imaging. a) Images from a continuously immobilized *C. elegans*, showing an ALM neuron severed 20 µm from the cell soma (top panel) immediately following laser axotomy and (bottom panel) after 10 hr. Arrow indicates lesion point. b) Average regenerative outgrowth of ALM neurons severed at the indicated distances. Outgrowth was measured from images taken at 10 hr post surgery and categorized as initiating from the lesion point or from the cell soma (* indicates p<0.05 by Student’s t-test). c) Average FRET signals measuring cellular calcium response to laser axotomy. At each time point, two channel fluorescent FRET images of the target neuron where averaged across the cell soma and along the axon within 5 µm of the lesion point to measure intracellular calcium levels in those regions. Arrows indicate time of laser damage (t = 0 s). Shaded regions indicate standard error on the mean at each time point. n indicates number of worms assayed.

We used a laser to sever the process of the left or right ALM neuron at specific distances from the cell soma: 10 µm, 20 µm or 40 µm (Fig. 5a). Using 10% agarose and the additional wax stabilization technique described in the methods, worms remained immobile for more than 10 hr after surgery, allowing automated imaging of the subsequent regeneration (Movie S1). Previous studies have shown that axotomy induces axonal regrowth not only at the cut site but can also result in new outgrowth at the cell body [12]. Images were analyzed to measure the length of regenerative outgrowth initiating from the region of the laser lesion vs. the cell soma (Fig. 5b). We found that cuts at all distances induce a similar amount of regrowth at the cut site. However, we found that cuts at 40 µm from the cell soma led to reduced regeneration at the cell body relative to cuts at 10 µm or 20 µm. This result suggests that the axotomy-induced regeneration signal within the cell is spatially localized to some degree.

In a parallel series of experiments, we performed axotomies on ALM neurons expressing the calcium sensitive fluorescent reporter YC3.60 [20]. We then acquired images every 3 sec for 5.5 min. We analyzed images to measure the calcium signaling in (1) the cell soma and (2) within 5 µm of the lesion point (Fig. 5c). This analysis again required sub-micron stability of the target neuron throughout the imaging period. We found that calcium response within the cell soma decreased dramatically with distance between the soma and cut location. These results suggest that the differential activation of the calcium response may contribute to the different degrees of regeneration in the soma and axon.

## Discussion and Conclusions

Our immobilization method using polystyrene nanoparticles and agarose pads is a simple and versatile technique for long-term imaging of larval and adult C. *elegans* without anesthetic compounds. We have used our method to investigate calcium transients and axonal regrowth following laser surgery in immobilized animals. Our techniques enable new studies on dynamic cellular processes within an intact physiological context. Moreover, combined with the powerful genetic methods afforded by *C. elegans*, our techniques will allow simple, rapid and affordable *in vivo* time-lapse analysis across relevant genetic backgrounds. In addition to the applications described here, we have used our method for laser ablation of nuclei, imaging of embryogenesis, and calcium imaging of the temperature sensory system.

Our results support a model for immobilization in which polystyrene particles increase the friction between the worm and the solid surfaces with which it is in contact. Such a model is consistent with a microscopic description of friction [21]. The static friction force between two surfaces is equal to the contact area multiplied by the interfacial shear strength. As the pressure between the worm and the pad increases, both the contact area and the interfacial shear strength may increase, thereby increasing the friction. The large surface area of small diameter polystyrene beads may play a role in increasing both contact area and the interfacial shear strength relative to the case without beads. Consistent with these ideas, the highest degree of immobilization occurs when the smallest (0.05 µm diameter) nanoparticles are used in conjunction with a high concentration (10%) agarose pad. However, immobilization on high concentration agarose pads causes greater compression of the worm. We suggest selecting the agarose concentration based on the minimum required to achieve an acceptable level of immobilization for the phenomenon being studied.

A potential concern with our method is that the animals do not feed while immobilized. Our immobilization method completely inhibits pharyngeal pumping, possibly due to increased mechanical stimulation [22] from compression against the agarose pad. It may be possible to maintain feeding during immobilization by the addition of serotonin to the pad.

One limitation of our method compared to gluing or some microfluidic immobilization methods is that the worm is not easily accessible for delivery of mechanical or chemical stimuli, or contact by electrodes. In addition, while our method allows parallel immobilization of multiple worms, it is not well suited for serially imaging or sorting a large number of animals. However, for applications in which these limitations are not of significant concern, we expect our method to be widely useful.

## Supporting Information

Movie S1 **Time-lapse movie of regeneration of an ALM neurite in a nanoparticle-immobilized worm.**
(MOV)
